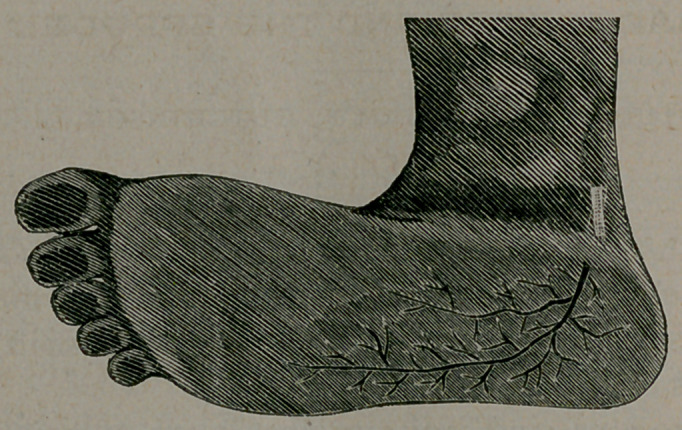# Plantar Neuritis; Resection of Plantar Nerve

**Published:** 1891-02

**Authors:** T. J. Bennett

**Affiliations:** Austin, Texas


For Daniel's Texas Medical Journal.

### A CASE OF PLANTAR NEURITIS WITH RESECTION OF THE PLANTAR CUTANEOUS NERVE; RECOVERY.

BY T. J. BENNETT, M. D., AUSTIN, TEXAS.

Read before the Austin District Medical Society, June 19, 1890.

THE PATIENT a negress, age about sixty years. Family history good. Sought advice for severe pains in the soles of the feet. An examination revealed tremendous callosities on the plantar surfaces of both feet,—that on the right being much the greater. This tissue, near the heel where it was thickest, was almost as hard as bone, and measured fully a quarter of an inch in depth. The greatest pain was located in the thickest part of the callous, and radiated along the nerve distributions, diminishing in severity towards the middle and phalangeal portion of the sole. The patient had suffered almost continuously, and at times excrutiatingly, for the last twelve or thirteen years.

The severset pains were felt in the evening, after the patient had been on her feet all day. No history of swelling of the feet, nor of gout, could be had. The patient had always been well, up to the first appearance of the present trouble. No relief, more than temporary, had ever been obtained from any treatment suggested.

Paring off the dense tissue and applying emolient dressings seemed to afford the greatest relief, but in a few days the callous had returned, and with it the pains, as severe as ever. The surface, after the compact tissue had been removed, was extremely sensitive and red, showing conclusively a local neuritis. The callous had incorporated the nerve filaments, and thus an inflammation had been produced, and the constant alternate pressure from walking had kept it up. What produced the callous was conjectural, though I may state that it has been a common observation to see the greater number of plantation negroes with immense callosities on the soles of their feet, due to their habit of going barefooted. This patient was reared on the farm, and it is very probable the horny layer of her sole was hypertrophied from this cause, though she denies ever having gone a day without her shoes. Her story about the appearance of the callous was that it, and the pains came on simultaneously, and rather suddenly.

It occurred to me that a resection of the nerve supplying the integument and fascia would probably afford relief. The plantar cutaneous nerve is one of the terminal branches of the posterior tibial, and supplies the plantar surface of the heel with sensation. It perforates the internal annular ligament near the tendo-achillis to the os calcis on a line from its inner malleolus. The dissection was made at this point, and about half an inch of the nerve was cut away, as it emerged through the ligament. The wound was dressed antiseptically, and united by first intention. Relief was prompt and complete, and up to this time, about five months, there has been no return of the pain.

The operation was painless. About ten minims of a four per cent. solution of cocaine was injected into the tissues at the site of the proposed dissection, and then an Esmarch's bandage applied. The latter, I am satisfied, greatly assisted the cocaine in rendering the operation painless.

---

Removals.—Dr. Z. T. Bundy has removed from Waxahachie to Milford, Ellis county; Dr. W. B. Gibson has removed from Austin to Sherman; Dr. W. L. Cooke from San Marcos to Wimberley's Mill, Hays county; Dr. T. W. Styles from Paige to Stephensville; Dr. S. H. Weatherford from Georgetown to Taylor; Dr. C. Hamilton from Dial, to Rome, Ga.

## Figures and Tables

**Figure f1:**